# Turpentine Vapor in Accidents from Chloroform Vapor

**Published:** 1879-01

**Authors:** 


Turpentine Vapor in Accidents from Chloroform Vapor.—Dr. Wachsmuth, of Berlin, has suggested a preventive of those accidents which frequently occur in the administration of chloroform to produce anæthesia. He says: It consists in the addition of one part of rectified oil of turpentine to five parts of chloroform. The oil of tupentine in vapor appears to exert a stimulating or life-giving effect on the lungs, and protects these organs from passing into that paralyzed state which seems to be produced by chloroform narcosis. Dr. Wachsmuth, while lying on a sick-bed, accidentally breathed the vapor of tupentine, and he experienced from this a strongly refreshing feeling. This fact induced him to try the plan of adding oil of turpentine to chloroform when the latter was used for anæsthetic purposes. The beneficial results surpassed his expectation.—*Boston Journal of Chemistry*, October, 1878.

